# Probing Gas Adsorption in Zeolites by Variable-Temperature IR Spectroscopy: An Overview of Current Research

**DOI:** 10.3390/molecules22091557

**Published:** 2017-09-15

**Authors:** Edoardo Garrone, Montserrat R. Delgado, Barbara Bonelli, Carlos O. Arean

**Affiliations:** 1 Politecnico di Torino, The Department of Applied Science And Technology and INSTM Unit of Torino-Politecnico, Corso Duca degli Abruzzi 24, 10129 Turin, Italy; d002696@polito.it (E.G.); barbara.bonelli@polito.it (B.B.); 2 Department of Chemistry, University of the Balearic Islands, E-07122 Palma, Spain; montserrat.rodriguez@uib.es

**Keywords:** dual sites, gas adsorption, IR spectroscopy, VTIR spectroscopy, zeolites

## Abstract

The current state of the art in the application of variable-temperature IR (VTIR) spectroscopy to the study of (i) adsorption sites in zeolites, including dual cation sites; (ii) the structure of adsorption complexes and (iii) gas-solid interaction energy is reviewed. The main focus is placed on the potential use of zeolites for gas separation, purification and transport, but possible extension to the field of heterogeneous catalysis is also envisaged. A critical comparison with classical IR spectroscopy and adsorption calorimetry shows that the main merits of VTIR spectroscopy are (i) its ability to provide simultaneously the spectroscopic signature of the adsorption complex and the standard enthalpy change involved in the adsorption process; and (ii) the enhanced potential of VTIR to be site specific in favorable cases.

## 1. Introduction

Owing to the regular layout of channel systems and adsorption sites that facilitates the tailored design of gas adsorption properties, periodic porous solids such as zeolites, metal-organic frameworks (MOFs) and related materials are increasingly being investigated as (potentially) improved adsorbents in a wide range of industrial gas separation and purification processes [1,2,3,4,5,6,7,8,9,10,11,12,13]; among them, CO_2_ capture from the flue gas of power stations burning fossil fuels, sweetening of natural gas, purification of syngas, petrol desulfurization and hydrogen separation from steam reforming of hydrocarbons, to quote only some examples. In several of such processes, the gas adsorbent units are commonly operated in a transient mode that involves alternating adsorption-desorption cycles referred to as pressure swing (PSA) or temperature swing adsorption (TSA), depending on the strategy being used for adsorbent regeneration. In either case, it should be clear that improvement of the adsorbent should be aimed at the double purpose of increasing (differential) gas adsorption capacity while keeping interaction energy (of the retained gas) small enough to curb the costs of adsorbent regeneration. To that endeavor, precise characterization of the adsorption sites and the determination of the gas-solid interaction modes and corresponding interaction energy are prime requirements. Moreover, a similar strategy can also be used in the search for cost-effective adsorbents for large-scale (reversible) storage and delivery of fuels such as methane and hydrogen; which constitutes a present-day strategic issue in the transportation sector [14,15,16,17,18,19,20]. Aside from the foregoing usage, increased knowledge of the nature and layout of active adsorption sites is also highly relevant to the wide-ranging industrial application of zeolites as catalysts in several fields, such as the petrochemical industry, methanol to olefin conversion, catalytic production of specialty chemicals [21,22,23,24,25,26,27] and CO_2_ methanation [28,29], to mention only a few examples.

Infrared spectroscopy at a constant temperature, using adsorbed probe molecules (such as CO, N_2_ and several others), is a main experimental technique frequently used to obtain valuable information on active surface sites, by looking at frequency shifts of meaningful vibrational modes and at the (relative) intensity of the corresponding IR absorption bands. Nevertheless, quantitative measurement of the gas-solid interaction energy is out of reach of this classical application of IR surface spectroscopy; which for that purpose, needs to be complemented with another technique allowing for precise measurement of the heat of adsorption; such as adsorption calorimetry, evaluation of the isosteric heat of adsorption, temperature programmed desorption or inverse gas chromatography. There is, however, a recent development of IR surface spectroscopy, termed variable-temperature IR (VTIR) spectroscopy [30], that has the potential to provide characterization of the gas adsorption complex (identifiable by its characteristic IR absorption bands) and simultaneous measurement of the corresponding gas-solid interaction energy from a series of spectra recorded over a temperature range.

The aim of this account is to show the practical use of the VTIR method as applied to the simultaneous determination of both the thermodynamics of the gas-solid adsorption process and the structure of the resultant adsorption complexes. For that purpose, first an abridged outline of the theoretical basis and experimental procedure is given. This is followed by analysis of selected case studies of CO and CO_2_ adsorption on zeolites, which typify different meaningful situations. Among them, due attention is given to adsorption sites involving more than one zeolite extra-framework cation, which can be highly relevant to both gas separation and heterogeneous catalysis alike.

## 2. Outline of the VTIR Method

Regarding adsorption thermodynamics, the starting point of the VTIR method is the classical van ’t Hoff equation:

[∂ln *K*/∂T]_p_ = Δ*H*^0^/*RT*² (1)

where *K* is the equilibrium constant of the process being considered. Assuming (as is usually done) that Δ*H*^0^ and Δ*S*^0^ are both temperature independent, the van ’t Hoff relationship becomes:

ln *K*(*T*) = (−Δ*H*^0^/*RT*) + (Δ*S*^0^/*R*) (2)

The central tenet of the VITR method is that *K* can be determined, at any given temperature, from the intensity of a characteristic IR absorption band (a measure of the coverage of the adsorbed species) and the corresponding equilibrium pressure.

Referring to adsorption on a single type of site, with the formation of 1:1 adsorption complexes, the theory of the VTIR method is as follows. Let S be the empty surface site and M the adsorbed molecule, and let Equation (3) represent the adsorption process:

S_(s)_ + M_(g)_**⇄** S − M_(ads)_ (3)

For an ideal system, the activity of the occupied sites is given by the corresponding coverage, *θ*, and that of the empty sites by 1 − *θ*, while the activity of molecules in the gas phase is given by the corresponding equilibrium pressure, *p*. This leads to the Langmuir Equation (4) below:

*θ* = *N*/*N*_M_ = *K*(*T*)*p*/[1 + *K*(*T*)*p*] (4)

where *N* is the number of adsorbed moles under a pressure *p* and *N*_M_ that at full coverage. Besides the ideality of the adsorbed phase, other assumptions are: (i) the validity of the van ’t Hoff integrated Equation (2) and (ii) the validity of the Lambert–Beer law: the intensity, *A*, of the characteristic IR absorption band being considered is proportional to the amount adsorbed,

*A* = *b* × *N* (5)

(where *b* is a proportionality constant).

Full coverage corresponds to the maximum intensity, *A*_M_, of the IR absorption band. The combination of the Equations (2), (4) and (5) leads to:

*θ* = (*A*/*A*_M_) = exp [Δ*S*^0^/*R*] exp [−Δ*H*^0^/*RT*] *p*/{1 + exp [Δ*S*^0^/*R*]exp[−Δ*H*^0^/*RT*] *p*} (6)

Equation (6) describes the expected temperature and pressure dependence of the intensity of the relevant IR absorption band, as a function of the parameters Δ*S*^0^, Δ*H*^0^ and *A*_M_. Details regarding the validity of the Langmuir adsorption model can be found elsewhere [30].

Two cases can occur when applying Equation (6). Either *A*_M_ is known independently, so that *θ* is directly measurable, or only an approximate value (e.g., a lower limit) of *A*_M_ is available. In the first case, Equation (6) becomes:

ln {*θ*/[(1 − *θ*) *p*]} = (Δ*S*^0^/*R*) − (Δ*H*^0^/*RT*) (7)

which gives direct access to both Δ*S*^0^ and Δ*H*^0^.

When *A*_M_ is not accurately known, Equation (6) may be written as:

ln {*A*/[(*A*_M_ − *A*) *p*]} = (Δ*S*^0^/*R*) − (Δ*H*^0^/*RT*) (8)

which includes *A*_M_ as a non-linear parameter, to be determined by an iteration procedure and linear regression of Equation (8), until the best fit is attained (see Section 3).

Some critical points involved in the VTIR method merit comment. First, use of the van ’t Hoff relationship for deriving thermodynamic quantities can have drawbacks when the temperature range covered is small, e.g., a spurious correlation between Δ*H*^0^ and Δ*S*^0^ can appear [31]. To avoid this problem, VTIR spectra should be recorded over a sufficiently wide temperature range. Secondly, the assumption that both Δ*H*^0^ and Δ*S*^0^ are temperature independent implies that Δ*c*_p_^0^ (the difference in specific heat at constant pressure between the gas phase and the adsorbed phase in standard conditions) is nil. Strictly speaking, this condition is never fulfilled, because the degrees of freedom of the molecule are not the same in both states. However, translational degrees of freedom in the gas phase are replaced by low-lying vibrational ones in the adsorbed state [32], and the adsorbed molecule usually retains some rotational freedom. Hence, Δ*c*_p_^0^ is expected to be much smaller than 3*R*, and inclusion of the corresponding correction would not affect significantly the final results. Nevertheless, it should still be kept in mind that both Δ*H*^0^ and Δ*S*^0^ are the values of thermodynamic quantities that are actually temperature dependent, averaged over the temperature range spanned by the VTIR spectroscopic measurement. It is therefore appropriate to refer to both Δ*H*^0^ and Δ*S*^0^ as the average temperature of measurements, *T*_M_. Accordingly, both the standard states, for the gas and the adsorbed phase, have to be related to *T*_M_. The determined value of Δ*S*^0^ depends also on pressure through the gas phase entropy: the reference state for the gas is usually taken as 1 mbar (or 1 Torr), representative of the pressure at which measurements are carried out. The reference state for the Langmuirian phase is instead always *θ* = 1. Finally, it should be clear from the above considerations that the temperature range over which measurements are run is determined by the compromise of: (i) being large enough to avoid artefacts in the application of the van ’t Hoff equation and (ii) narrow enough so that standard entropy and enthalpy changes may be considered as being constant.

**Figure 1:**
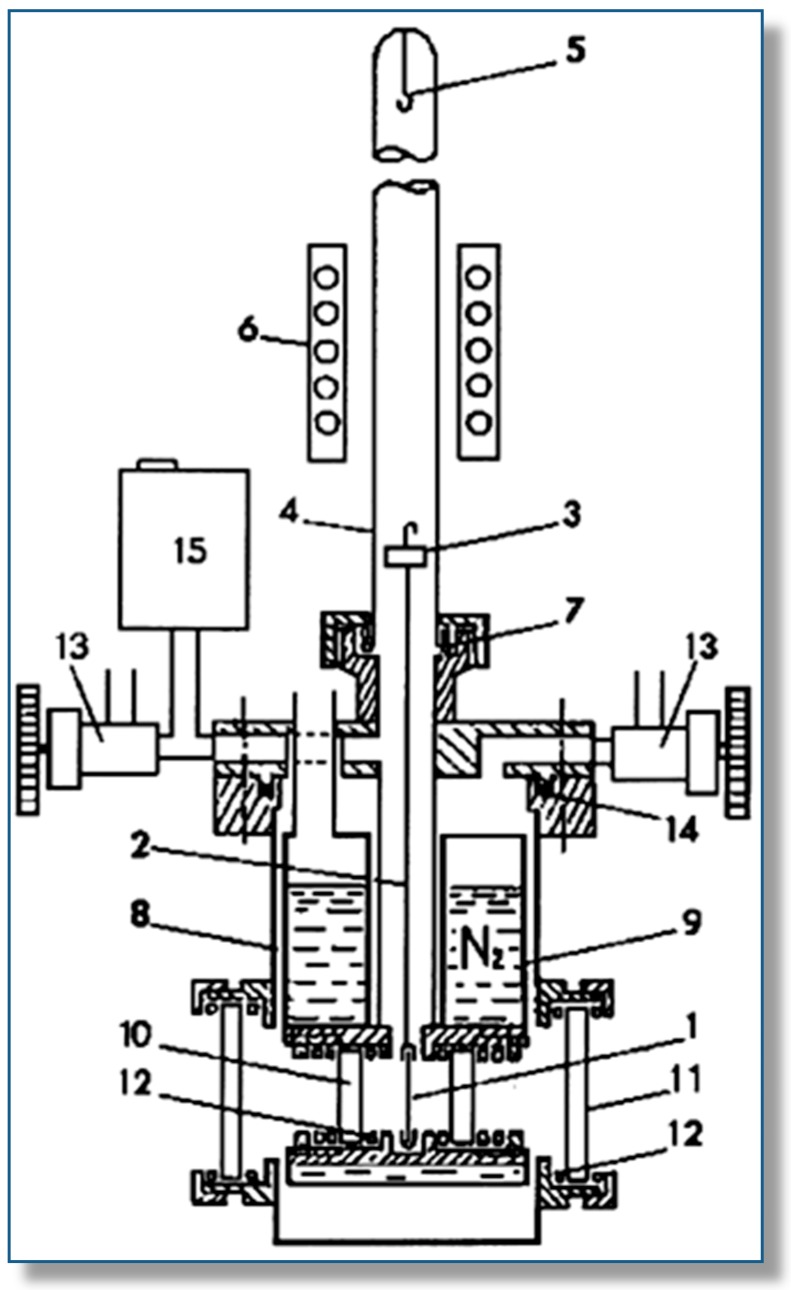
Scheme of the homemade (stainless-steel) variable-temperature IR cell: (1) sample wafer, (2) sample holder, (3) magnetically-driven anchoring piece, (4) quartz tube, (5) hook for fixing the sample wafer inside the furnace, (6) furnace, (7) Viton O-ring, (8) cell body, (9) refrigerated region, (10) and (11) optical windows, (12) indium gaskets, (13) valve, (14) Teflon gasket, (15) pressure gauge ([33]).

On the experimental side, measurement of VTIR spectra requires the use of a properly designed IR cell: some commercial cells are adaptable for such a purpose, but most of the results discussed below were obtained by using a homemade cell [33], which is depicted (schematically) in Figure 1; further details on cell design and operation can be found elsewhere [33,34]. Measurements are run by dosing a fixed amount of the adsorbate gas into the cooled IR cell, which contains the activated (outgassed) solid adsorbent wafer, after which the cell is closed, and a series of VTIR spectra is obtained at an increasing temperature, while simultaneously recording temperature and equilibrium pressure. Note that the cell is thus operated as a closed system in the thermodynamic sense; in contrast to calorimetric or volumetric adsorption measurements, which are usually performed in open systems. Further details on cell operation and sample preparation are given in Section 3.

## 3. Selected Case Studies

### 3.1. Carbon Dioxide Adsorption in H-MCM-22

Adsorption of CO_2_ in the protonic zeolite H-MCM-22 (Si:Al = 16:1) was recently studied by means of both VTIR spectroscopy and adsorption calorimetry [35]. Protonic zeolites contain the structural group [Si(OH)Al], where a proton is attached to an oxygen atom that bridges skeletal tetrahedrally-coordinated Si and Al atoms; and the characteristic O‒H stretching frequency of those hydroxyl groups changes when interacting (through hydrogen bonding) with adsorbed CO_2_. Since this interaction also brings about a shift of the asymmetric ν_3_ mode of the corresponding carbon dioxide molecule, the system CO_2_/H-MCM-22 affords an example in which both the adsorbed molecule and the adsorbing site can (in principle) be used for VTIR spectroscopic measurements. However, application of the VTIR method to the O‒H stretching band facilitates direct knowledge of coverage; because at any given temperature and gas equilibrium pressure, the fraction 1 − *θ* of empty adsorption sites can be directly obtained by dividing the corresponding OH band intensity by its maximum value, namely that shown by the blank zeolite spectrum. Hence, in principle, this system would seem to be particularly well suited to VTIR spectroscopic study.

**Figure 2:**
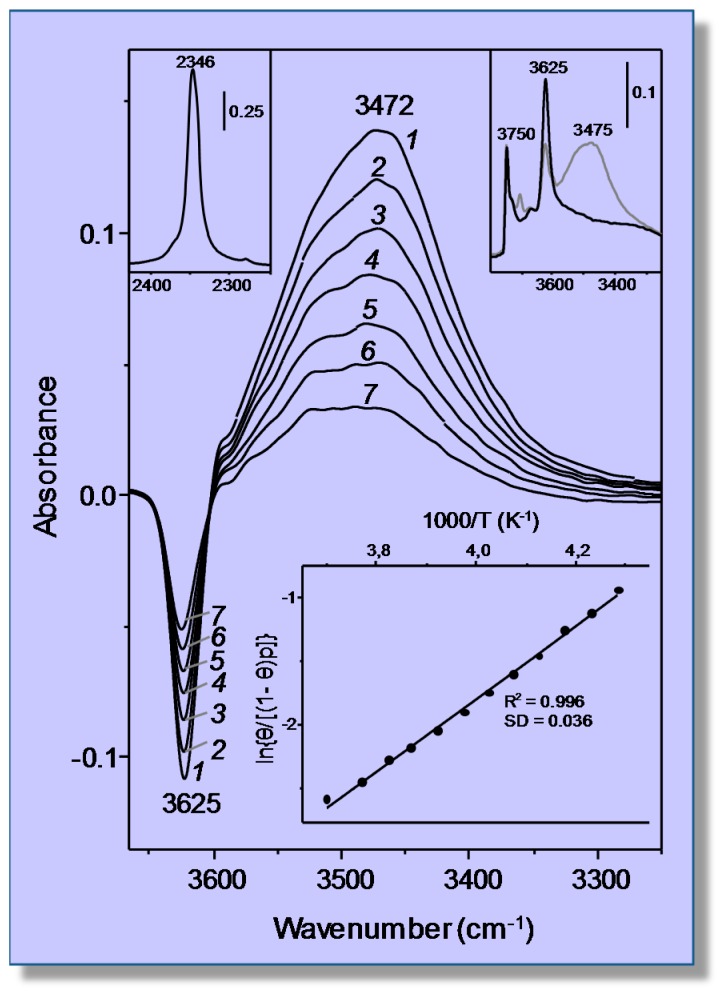
Representative variable-temperature IR spectra (O−H stretching region) of CO_2_ adsorbed on H-MCM-22. The spectra are shown in the difference mode (zeolite blank subtracted). From *1–7*, temperature goes from 233–275 K; and equilibrium pressure from 4.49–6.22 mbar. The top inset (right) shows the IR spectra in the O−H stretching region of the blank zeolite H-MCM-22 wafer (black line), and after dosing with CO_2_ at 77 K (gray line). The top inset (left) shows the asymmetric ν_3_(CO_2_) stretching region of Spectrum *1*. The bottom inset shows the van ’t Hoff plot for CO_2_ adsorbed on H-MCM-22: data obtained from the O−H stretching band at 3625 cm^−1^. R, linear regression coefficient; SD, standard deviation.

Figure 2, in the top inset on the right, shows the IR spectrum in the O‒H stretching region of the blank zeolite wafer at 77 K, after previous activation (outgassing) by heating at 673 K for 4 h under a dynamic vacuum (residual pressure smaller than 10^−4^ mbar). The spectrum displays characteristic IR absorption bands that peak at 3750 and at 3625 cm^−1^. These bands arise, respectively, from silanols (which are of no concern herein) and from the bridged Si(OH)Al hydroxyl groups that constitute the zeolite adsorbing sites. Representative VTIR spectra of adsorbed CO_2_ are depicted in Figure 2, which shows decreased intensity of the 3625 cm^−1^ absorption band to an extent that (for a fixed CO_2_ dose) is a function of temperature. At the same time, a new (and much broader) IR absorption band coming from end-on hydrogen bonded OH···OCO species [36] builds up. This new band depicts a maximum at about 3472 cm^−1^, but it also shows shoulders on both sides of that maximum, thus giving clear evidence that the O‒H stretching band at 3625 cm^−1^ consists of several individual components, each of them having a (slightly) different interaction energy with the adsorbed CO_2_ molecule. As a matter of fact, the presence of several components, arising from a variety of non-equivalent framework oxygen atoms of the Si(OH)Al groups [37], was already shown some time ago by Onida et al. [38]. Nevertheless, our attempts at band resolution did not give quantitatively reliable results; mainly due to inherent uncertainty about how the band should be decomposed. Therefore, VTIR calculations were performed using the integrated intensity of the band envelope as it appears in the spectra shown in Figure 2. Note that the IR absorption band corresponding to the ν_3_ stretching mode of the adsorbed CO_2_ molecule (shown in the top inset on the left of Figure 2) is even less reliable regarding band resolution. From the whole series of VTIR spectra recorded (which covered the temperature range of 233–275 K), the linear plot shown in the bottom inset of Figure 2 was obtained, which rendered the value of Δ*H*^0^ = −24 kJ mol^−1^ for the standard enthalpy of adsorption of carbon dioxide in H-MCM-22; the estimated error limit being ± 2 kJ mol^−1^.

**Figure 3:**
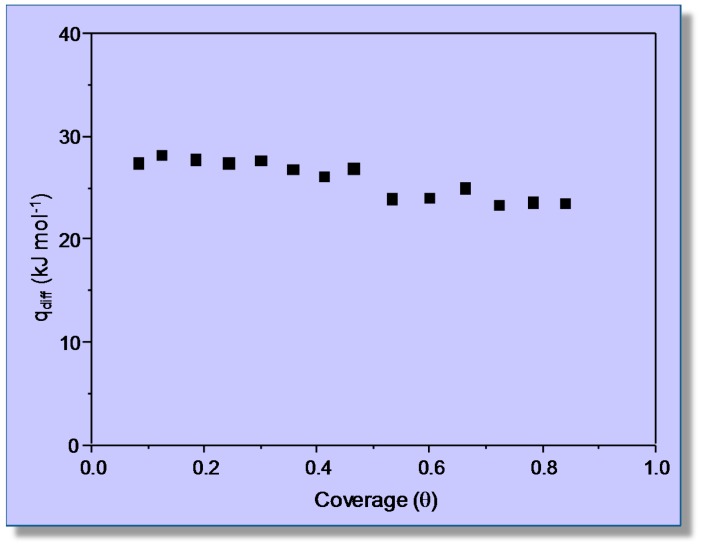
Adsorption heat of CO_2_ on H-MCM-22, at 303 K, as a function of coverage [35].

For comparison, calorimetric measurements of CO_2_ adsorption in the same zeolite sample were performed, at the constant temperature of 303 K, by using a Tian–Calvet-type microcalorimeter (Seratam BT2.15) connected to a purpose-built volumetric apparatus and following the procedure described in detail elsewhere [35]. Figure 3 displays the obtained values of differential heat of adsorption as a function of coverage, which were recorded allowing the system to equilibrate for 50 minutes after each CO_2_ dose. The average of these results gives an adsorption heat of about 26 kJ mol^−1^, which is in fairly good agreement with the value of Δ*H*^0^ = −24 kJ mol^−1^ obtained by the VTIR spectroscopic measurements. In both cases, the reported results correspond to an averaged interaction energy of the adsorbed CO_2_ molecules with the OH adsorption sites of the H-MCM-22 zeolite; no resolution into individual (homogeneous) groups of hydroxyl adsorption sites was attained.

### 3.2. Carbon Monoxide Adsorption in the Alkaline Zeolite Li-ZSM-5

Homo-cationic zeolites often show different cation sites that are not equivalent regarding the interaction with adsorbates. As an example, we will consider carbon monoxide adsorption in the alkaline zeolite Li-ZSM-5. As a matter of fact, IR spectroscopy of the CO/Li-ZSM-5 system has been reported in the literature several times, but to the best of our knowledge, a VTIR spectroscopic study (including gas-solid interaction energy) has not been as yet reported. For VTIR spectroscopy, a thin self-supported wafer of the zeolite sample (which had a Si:Al ratio of 30:1) was prepared and activated (outgassed) in a dynamic vacuum (residual pressure <10^−4^ mbar) for 4 h at 700 K inside the IR cell. Liquid nitrogen was used for cooling, and in order to optimize thermal contact between the sample wafer and the cool cell body, 0.2 mbar of helium was admitted into the sample compartment before the background spectrum at 77 K was recorded. The cell was then dosed with CO and closed, and IR spectra were recorded at several temperature values (within the range of 234–282 K) upon gradual warming of the IR cell. Simultaneously, temperature and equilibrium pressure inside the sample compartment were recorded. For that purpose, a platinum resistance thermometer (Tinsley, London, UK) and a capacitance pressure gauge (MKS, Baratron) were used. The precision of these measurements was better than ±2 K and ±10^−2^ mbar, respectively. Pressure correction (for helium) was determined from a previous calibration plot. Care was taken to run a previous CO adsorption isotherm (increasing CO doses at 77 K) in order to (i) determine an approximate value of *A*_M_ and (ii) choose for the VTIR measurements a CO dose small enough to avoid the formation of Li(CO)_2_^+^ dicarbonyl species [39,40], which would give an IR absorption band partially overlapping those of monocarbonyl (Li^+^···CO) adducts, thus complicating precise measurement of their corresponding band intensity. Transmission FTIR spectra were recorded at 2-cm^−1^ resolution on a Bruker Vertex 80v instrument (Billerica, MA, USA); 64 scans were accumulated for each spectrum.

**Figure 4:**
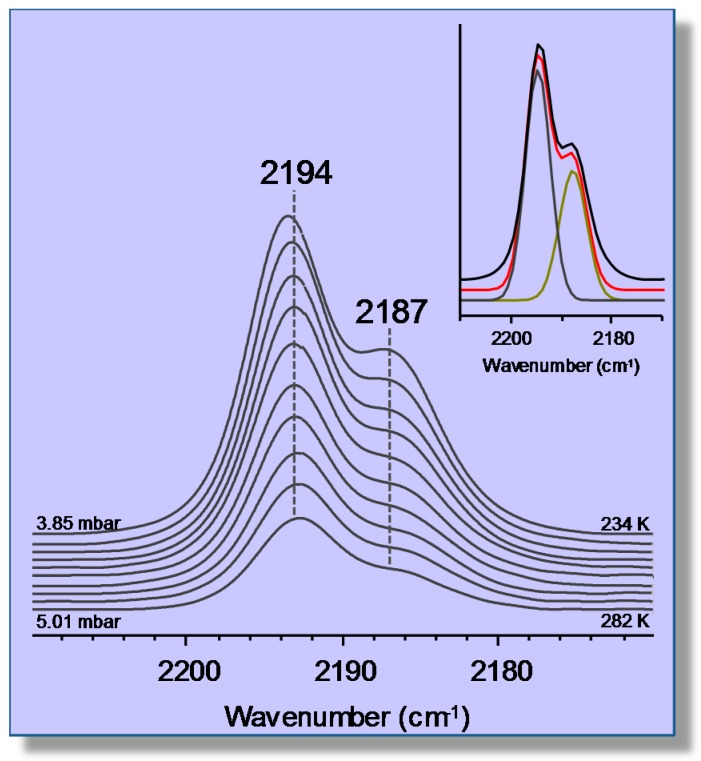
Representative variable-temperature IR spectra of CO adsorbed on Li-ZSM-5 (Si:Al = 30:1). From top to bottom, the temperature increases from 234–282 K; and equilibrium pressure from 3.85–5.01 mbar. The inset shows an example of the band resolution.

**Figure 5:**
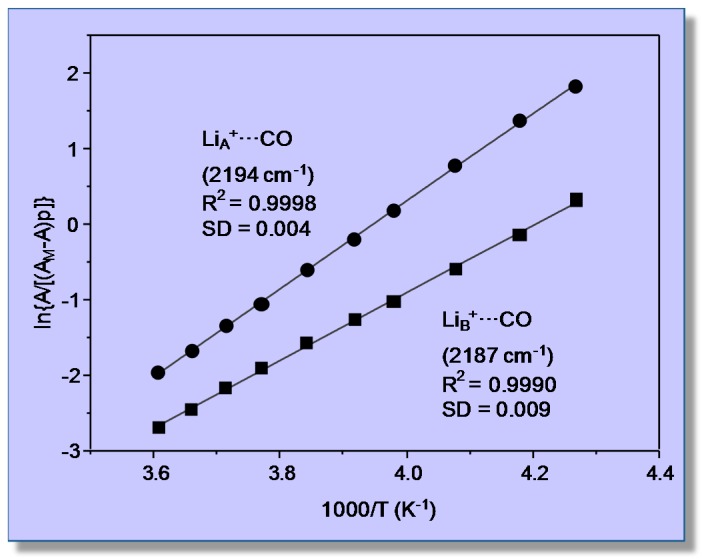
Van ’t Hoff plots for CO adsorbed on Li-ZSM-5 (Si:Al = 30:1); data obtained from the IR absorption bands at 2194 (Li_A_^+^···CO) and 2187 cm^−1^ (Li_B_^+^···CO). R, linear regression coefficient; SD, standard deviation.

Variable-temperature FTIR spectra (in the C‒O stretching region) of adsorbed carbon monoxide are shown in Figure 4. Two (partially overlapping) IR absorption bands are seen at 2194 and 2187 cm^−1^; as expected, their intensity decreases as temperature is increased, but the peak wavenumbers remain constant, which constitutes strong evidence that no dicarbonyl species are formed. According to previous reports [41,42], these IR absorption bands are assigned to monocarbonyl Li^+^···CO species formed on two types of Li^+^ sites, which will be termed hereafter Li_A_ (IR absorption band at 2194 cm^−1^) and Li_B_ (band at 2187 cm^−1^), respectively. The corresponding values of integrated band intensity obtained after computer resolution of the variable-temperature IR spectra displayed in Figure 4 were used to obtain the linear van ’t Hoff plots depicted in Figure 5. The needed values of *A*_M_ were refined by an iteration procedure involving small changes of integrated absorbance (around the approximate *A*_M_ value experimentally determined) until the best linear fit of Equation (8) for all of the experimental data (corresponding to each IR absorption band) was obtained. From the refined values of *A*_M_, it was inferred that the experimental points in Figure 5 correspond to a coverage 0.1 ≤ *θ* ≤ 0.5 for both types of Li^+^ sites.

**Figure 6:**
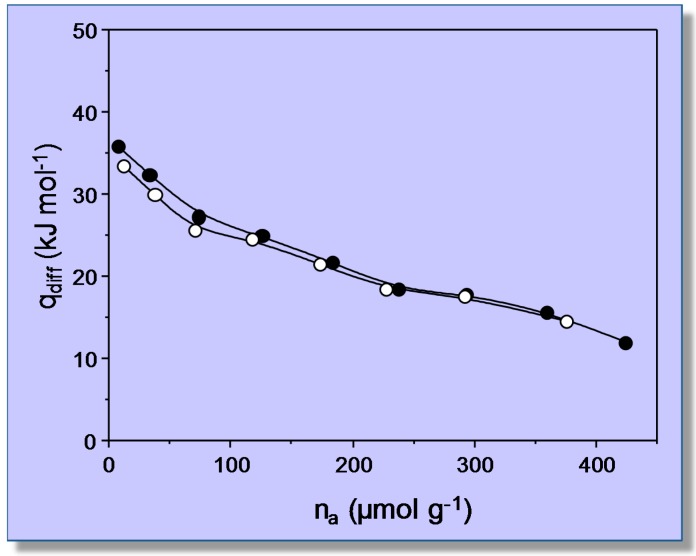
Differential heat of adsorption of CO on Li-ZSM-5 at 303 K as a function of adsorbed amount. Black symbols: primary isotherm; empty symbols: secondary isotherm.

The linear plots shown in Figure 5 rendered the value of Δ*H*^0^ = −44 (±2) kJ mol^−1^ for CO adsorption on Li_A_ sites, while the corresponding value for CO adsorption on Li_B_ sites resulted in being Δ*H*^0^ = −33 (±2) kJ mol^−1^. Regarding the nature of these two types of lithium sites, theoretical calculations have shown that Li_A_ sites are located at intersections of straight with zigzag channels of the zeolite, while Li_B_ sites are situated on the wall of both types of channels [41,42]. At channel-intersection sites, the Li^+^ ion is coordinated to only two oxygen atoms of the zeolite framework, while it is coordinated to three or four (depending on the specific location being considered) framework oxygen atoms when situated on channel-wall sites. Hence, the polarizing power of Li^+^ ions at Li_A_ sites should be greater than that of Li^+^ ions at Li_B_ sites, and this explains the stronger interaction energy with the adsorbed CO molecule (IR absorption band at 2194 cm^−1^) in the first case and the weaker interaction (IR absorption band at 2187 cm^−1^) in the second. Moreover, Grajciar et al. [43] have recently studied CO adsorption in Li-ZSM-5 by means of dispersion-corrected DFT calculations, arriving at −Δ*H*^0^ values in the range of 42–48 kJ mol^−1^ for adsorption on channel-intersection sites and 34–42 kJ mol^−1^ in the case of channel-wall sites. These results are in fairly good agreement with those of 44 (±2) and 33 (±2) kJ mol^−1^, respectively, reported herein. By contrast, a previous calorimetric measurement [39] gave the result shown in Figure 6. The extrapolated value of adsorption heat at zero coverage (about 36 kJ mol^−1^) is not far from the higher Δ*H*^0^ value obtained by using VTIR spectroscopy; but it should be acknowledged that, in this case, VTIR provides a more clear-cut discrimination between the two types of Li^+^ sites than what calorimetry (on its own) can give.

## 4. Adsorption Sites Involving More Than One Cation

### 4.1. Carbon Monoxide Adsorption on Na-FER

Small molecules from the gas phase are adsorbed inside the pore system of zeolites by interaction with specific adsorption sites, which are frequently considered to consist of an under-coordinated (extra-framework) metal cation and the nearby framework oxygen atoms. Therefore, most of the research work reported in the literature so far was focused on the interaction between those single cation sites and the adsorbed molecule. Nevertheless, ongoing research that combines VTIR spectroscopy with periodic DFT calculations has clearly shown that the gas adsorption site can actually involve two (or more) cations [44], which can be bridged by the adsorbed molecule giving rise to dual (or multiple) cation sites. Moreover, the bridged adsorption complex is usually more stable than that formed at a single cation site [44,45]; a fact that can be exploited for increasing selective adsorption of the desired individual component in a gas mixture. With a view toward highlighting the valuable new insights that can be obtained by exploring this field with VTIR spectroscopy, we report below (as an example) an abridged account of research work concerning the investigation of cationic adsorption sites in the zeolite Na-FER by using CO as a probe molecule. It is important to note that carbon monoxide has been (for many years) a widely-used probe molecule for IR spectroscopic studies of both zeolites and metal oxides [46,47,48,49,50,51], but every now and then, problems appeared when trying to assign some IR absorption bands without invoking the concept of dual cation sites [52,53,54].

**Figure 7:**
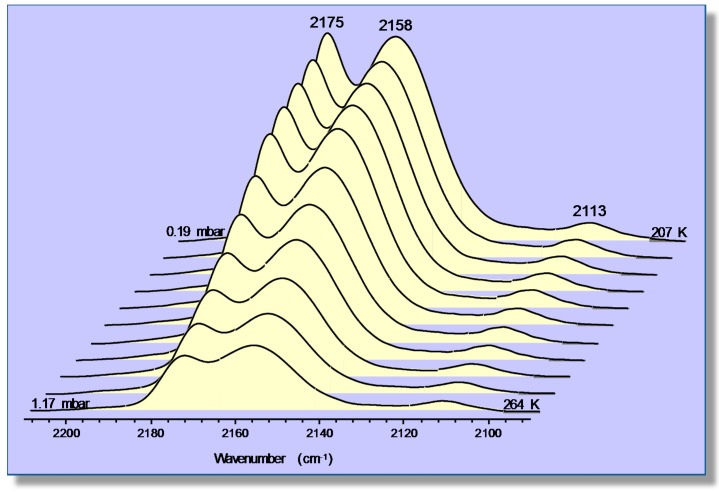
VTIR spectra (zeolite blank subtracted) of CO adsorbed on Na-FER, Si:Al = 8:1. Temperature increasing from 207–264 K; and equilibrium pressure from 0.19–1.17 mbar (from back to front).

**Figure 8:**
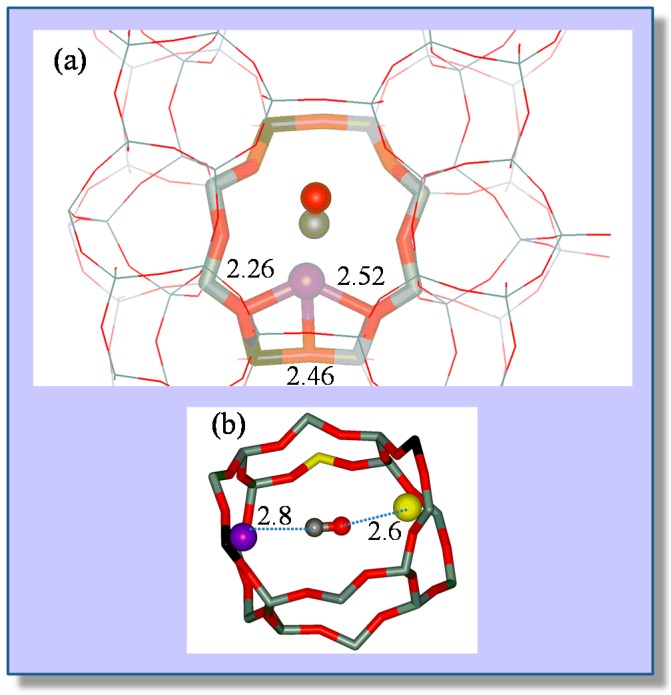
(**a**) Coordination of the Na^+^ cation and corresponding CO adsorption complex in a single cation site of Na-FER. Framework Al, Si and O atoms are depicted in black, grey and red colors, respectively; Na^+^, C and O atoms of CO are shown as violet, grey and red balls, respectively. The distances between Na^+^ and the nearest framework O atoms are given in Å; (**b**) CO adsorption complex on a dual cation site in Na-FER. The CO molecule interacts with the primary Na^+^ cation (violet ball) through the C atom (grey ball) and with the secondary Na^+^ cation (yellow ball) through the O atom (red ball). For further details, see [44].

**Figure 9:**
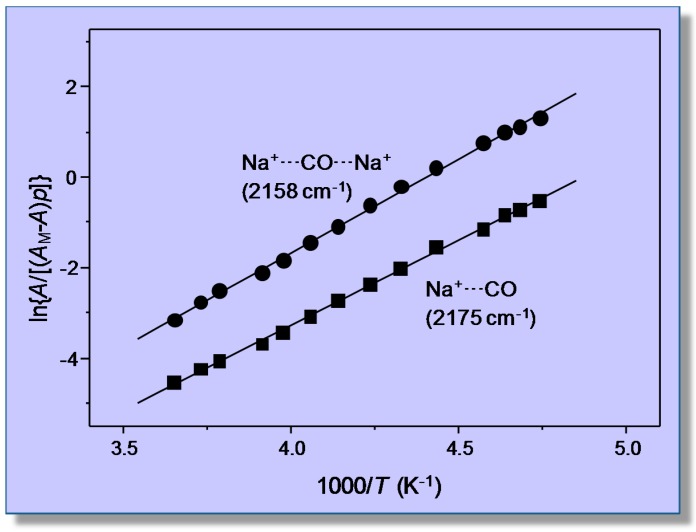
Van ’t Hoff plots for CO adsorbed on Na-FER, data obtained from the IR absorption bands at 2158 cm^−1^ (Na^+^···CO···Na^+^) and at 2175 cm^−1^ (Na^+^···CO).

Representative VTIR spectra of CO adsorbed on Na-FER (Si:Al ratio 8:1) are depicted in Figure 7; they correspond to a surface coverage range 0.05 ≤ θ ≤ 0.25. Two distinctive IR absorption bands are seen at 2175 and 2158 cm^−1^, along with a much weaker one at 2113 cm^−1^. According to theoretical DFT calculations [55], the band peaking at 2175 cm^−1^ should correspond to the C‒O stretching mode of carbon monoxide C-bonded to Na^+^ ions located in single cation sites, as shown in Figure 8a (calculated ν_(CO)_ values, 2174–2178 cm^−1^), while that at 2158 cm^−1^ should be assigned to CO molecules bridging two Na^+^ ions in dual-cation sites, depicted in Figure 8b (calculated ν_(CO)_ values, 2153–2175 cm^−1^). The weak IR absorption band at 2113 cm^−1^ corresponds to O-bonded adsorption complexes formed by a small fraction of CO molecules interacting through the oxygen atom with single-site Na^+^ ions and which are in a temperature-dependent equilibrium with the corresponding C-bonded species [56,57]. These O-bonded (Na+···OC) adsorption complexes were discussed in detail elsewhere [55], and no further consideration will be given to them herein. After computer resolution of the IR absorption bands at 2175 and 2158 cm^−1^ (Figure 7), the linear van ’t Hoff plots shown in Figure 9 were obtained. From those linear plots, the corresponding standard adsorption enthalpy values resulted in being Δ*H*^0^ = −30.5 (±2) kJ mol^−1^ and Δ*H*^0^ = −34.6 (±2) kJ mol^−1^ for CO adsorption at single and dual cation sites respectively; which coincide, within experimental error, with the corresponding calculated values of −29 and from −32 to −35 kJ mol^−1^, respectively [55].

**Figure 10:**
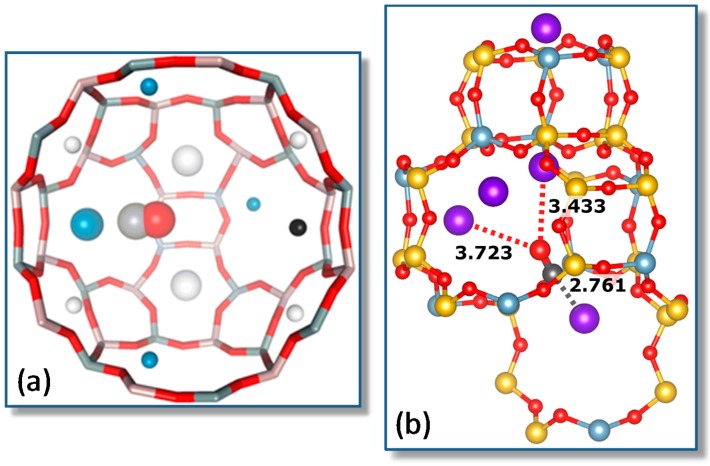
(**a**) T-shaped CO adsorption complex formed on the multiple cation site S2-(S1,S1) in Na-A. C and O atoms of the CO molecule are shown as grey and red spheres, respectively. Na^+^ cations in S1, S2 and S3 are depicted as white, blue and black balls, respectively; Na^+^ cations close to the CO molecule are shown as larger spheres; (**b**) Structure of a CO adsorption complex involving three cations in Na-CHA. Distances between primary and secondary cations and the CO molecule are given in Å. For details, see [59,60].

Again, it should be clear from this case study that a main advantage of the VTIR technique over both standard adsorption calorimetry and determination of the isosteric heat of adsorption is that VTIR can be site specific. Note that the small difference (4 kJ mol^−1^) between the Δ*H*^0^ values corresponding to Na^+^···CO adsorption complexes on single sites and Na^+^···CO···Na^+^ complexes on dual cation sites implies that both site types adsorb CO simultaneously, as actually shown by the IR spectra in Figure 7; and that would render calorimetry rather imprecise (if not impracticable) when trying to determine site-specific Δ*H*^0^ values, as already shown in the case of the CO/Li-ZSM-5 system. Moreover, the VTIR technique provides also the spectroscopic signature of each adsorption complex, which facilitates synergy that can be very fruitful when combining VTIR spectroscopy with periodic DFT calculations, aiming at detailed characterization of both solid adsorbents and adsorbed species. In fact, this combined theoretical and experimental approach has already been fruitfully used to investigate single and dual cation sites for CO adsorption on other high-silica zeolites; among them K-FER [45], Na-ZSM-5 and K-ZSM-5 [58]. Stabilization of the bridged CO adsorption complex on a dual cation site depends critically on the inter-cation distance; the optimum value being about 6.6 and 7.9 Å for Na^+^ and K^+^, respectively. A more complex situation can be found when dealing with alkaline zeolites having a small unit cell and low Si:Al ratio, such as the zeolite Na-A (LTA structure type) [59] and chabazite [60]. In such a case, the small volume of internal cages, together with the high concentration of extra-framework cations, tends to favor simultaneous interaction of the adsorbed CO molecule with more than two cations; usually by coordinating one (primary) cation through the carbon atom while interacting through the oxygen atom with two (secondary) cations, as shown in Figure 10 for the case of Na-A (Si:Al = 1:1) and Na-CHA (Si:Al = 2.6:1) [59,60].

### 4.2. Carbon Dioxide Adsorption in K-FER

**Figure 11:**
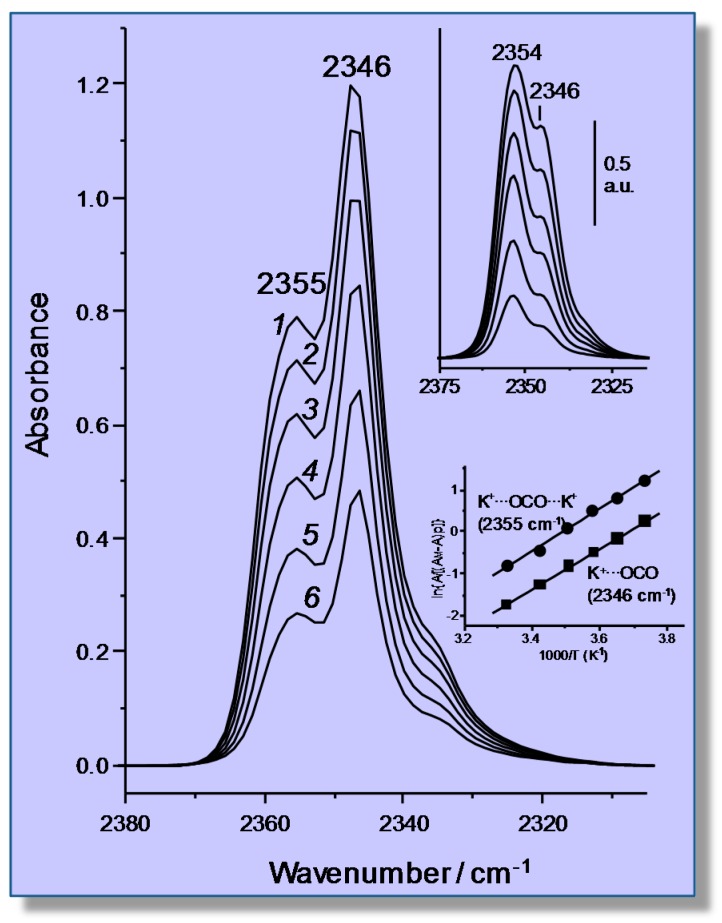
Representative variable-temperature IR (VTIR) spectra (ν_3_ region) of CO_2_ adsorbed on K-FER (Si:Al = 27.5:1). From *1*–*6*, the temperature goes from 268–301 K; and equilibrium pressure from 0.21–0.50 mbar. The zeolite blank spectrum was subtracted. The top inset shows the IR spectra of increasing doses of CO_2_ adsorbed on K-FER (Si:Al = 8.6:1) at room temperature. The bottom inset shows van ’t Hoff plots obtained for IR absorption bands arising from the single site K^+^···OCO (band at 2346 cm^−1^) and the dual site K^+^···OCO···K^+^ (band at 2355 cm^−1^) adsorption complexes.

As an example of single and dual cation sites for adsorbates other than CO, the case of carbon dioxide adsorption on a K-FER zeolite will now be briefly considered; full details can be found elsewhere [61]. Representative VTIR spectra in the ν_3_ region (asymmetric stretching) of CO_2_ adsorbed on K-FER (Si:Al = 27.5:1) are shown in the main body of Figure 11. Clearly, the two main IR absorption bands peaking at 2355 and 2346 cm^−1^, respectively, should correspond to two different types of CO_2_ adsorption complexes. In order to obtain complementary information that could help with understanding, an isothermal (room temperature) series of IR spectra at increasing equilibrium pressure was also run on other K-FER sample having a Si:Al ratio of 8.6:1, hence a much higher concentration of extra-framework cations. The results obtained are shown in the top inset of Figure 11, where it is seen that the lower Si:Al ratio results in an increased intensity of the IR absorption band at 2355 cm^−1^, as compared to that at 2346 cm^−1^; and this provides strong evidence that the former band should come from K^+^···OCO···K^+^ adsorption complexes bridging two K^+^ ions (dual sites), while that at 2346 cm^−1^ should correspond to K^+^···OCO species formed on single sites (which are expected to show a definite preponderance in the high silica zeolite). Further confirmation of those assignments was obtained by DFT calculations [61]; and analogous single and dual cation sites were also reported for the CO_2_/Na-FER system [62].

The bottom inset of Figure 11 shows the van ’t Hoff plots obtained after computer resolution of the two main IR absorption bands in Figure 11 (main body). From these linear plots, the Δ*H*^0^ values of −40 (±2) and −43 (±2) kJ mol^−1^ were found for the formation of the CO_2_ adsorbed species K^+^···OCO and K^+^···OCO···K^+^, respectively, which are both significantly higher than that of Δ*H*^0^ = −24 kJ mol^−1^ found for CO_2_ adsorption in H-MCM-22 (Section 3.1). It is also relevant to add that the experimentally (VTIR) found value of adsorption enthalpy of CO_2_ on H-FER was Δ*H*^0^ = −30 kJ mol^−1^; one half of which was computationally found to come from dispersion interactions [36].

## 5. Summary and Conclusions

Detailed understanding of gas adsorption on zeolites and related microporous materials is highly relevant to such fields as gas separation, purification and transport, gas sensing and heterogeneous catalysis, to quote only three main technological applications; hence the relevance to improve enabling techniques to probe both the energetics of the gas adsorption process and the structure of the adsorbed species. The foregoing discussion of selected case studies should help to appreciate how, for that purpose, VTIR spectroscopy has a clear edge over more classical methods such as adsorption calorimetry or the determination of the isosteric heat of adsorption: first, because VTIR can probe simultaneously the structure of the gas adsorption complex and the gas-solid interaction energy; secondly, because of the potential of VTIR spectroscopy to give (in favorable cases) site-specific information, which could not be available from calorimetry. Full exploitation of the VTIR method does depend, however, on (i) the intrinsic details of the gas-solid system being considered and (ii) the proper design of the experimental measurements. Thus, in the case of the CO_2_/H-MCM-22 system (considered in Section 3.1), neither calorimetry, nor VTIR spectroscopy afforded discrimination among several of the Brønsted acid OH adsorption sites known to be present in the adsorbent. By contrast, in the CO/Li-ZSM-5 system (Section 3.2), a difference of 10 kJ mol^−1^ in the corresponding adsorption enthalpy value was enough to discriminate between channel-intersection and channel-wall sites by VTIR spectroscopy; but not so by adsorption calorimetry on its own. Moreover, Δ*H*^0^ differences as small as 3–5 kJ mol^−1^ resulted in being good enough to discriminate between single and dual cation sites in the CO/Na-FER and CO_2_/K-FER systems (Section 4.1 and Section 4.2). Note that a clear-cut distinction between sites having such a close values of adsorption heat would be hard to obtain by calorimetric measurements.

In addition to alkaline zeolites considered above, it is noteworthy that precise identification of cation sites in zeolites other than alkaline is of the utmost importance regarding their use in several catalytic processes. For instance, Kuroda et al. [63,64] have recently reported on activation of ethane, at ambient temperature, by Cu-ZSM-5; and gave strong spectroscopic evidence showing that the catalytic center involves a dual-Cu^+^ adsorption site. A second example worth considering is the possibility of methane-to-methanol conversion by selective oxidation under mild conditions, so as to replace the current industrial process, which involves an energy-intensive first step of steam reforming of methane (to obtain syngas). Actually, in nature, methanotrophic bacteria do express methane monooxygenase (a kind of metalloenzyme containing dicopper or diiron active sites) to convert methane into methanol at ambient temperature, despite the high energy needed (435 kJ mol^−1^) to split the C-H bond [65,66,67]. It is remarkable that ongoing research carried out by several research groups (see, for instance, [68,69,70,71]) has shown that several copper-exchange zeolites can convert methane into methanol at a relatively mild temperature (150–200 °C), and there is evidence that in Cu-ZSM-5, the catalytic center involves a μ-oxo dicopper complex [72,73]; however, the mechanistic details are yet to be elucidated. Finally, we wish to mention earlier work by several authors [74,75,76] showing that Cu-ZSM-5 adsorbs dinitrogen-forming bridged Cu^+^···N-N···Cu^+^ complexes on dual Cu^+^ sites. Moreover, the same dual sites were found to be catalytically active in the decomposition of nitrogen oxides. We hope this abridged account of some specific cases can highlight the relevance of dual cation sites in catalysis.

To conclude, it should be clear that exploitation of the full potential of zeolites in several technological fields leans heavily on precise characterization of (i) their adsorption and their catalytically active sites, (ii) the thermodynamics of the adsorption process and (iii) the nature, structure and stability of adsorbed species. To that endeavor, several instrumental techniques, as well as theoretical calculations can be used; quite often in a synergistic combination. Hopefully, the critical appraisal made herein can contribute to clarify the merits, and limitations, of variable-temperature IR spectroscopy.
